# Differential Cell Death Pathways Induced by Oxidative Stress in Multi-Organs of Amur Grayling (*Thymallus grubii*) Under Gradient Ammonia Stress

**DOI:** 10.3390/antiox14040499

**Published:** 2025-04-21

**Authors:** Cunhua Zhai, Yutao Li, Ruoyu Wang, Ying Zhang, Bo Ma

**Affiliations:** 1 Heilongjiang River Fishery Research Institute, Chinese Academy of Fishery Sciences, Harbin 150070, Chinaliyutao@neau.edu.cn (Y.L.); wangruoyu@hrfri.ac.cn (R.W.); 2 College of Life Science and Technology, Harbin Normal University, Harbin 150025, China; 3 Key Laboratory of Cold Water Fish Germplasm Resources and Multiplication and Cultivation of Heilongjiang Province, Heilongjiang River Fishery Research Institute, Chinese Academy of Fishery Sciences, Harbin 150070, China; 4 National Agricultural Experimental Station for Fishery Resources and Environment, Fuyuan, Ministry of Agriculture, Harbin 150070, China; 5 Scientific Observation Station of Fisheries Resource and Environment in Heilongjiang River Basin, Ministry of Agriculture, Harbin 150070, China

**Keywords:** ammonia, *Thymallus grubii*, oxidative stress, multi-omics, intestinal microbe, cell death

## Abstract

Ammonia nitrogen is a common contaminant in aquatic environments, and its potential toxicity to organisms has attracted extensive attention. However, few studies have comprehensively evaluated the negative impacts of ammonia stress on cold-water fish. In this study, liver, gill, and intestine specimens of Amur grayling (*Thymallus grubii*) from three treatment groups (control (0 mg/L), low ammonia (43.683 mg/L), and high ammonia (436.8 mg/L)), were collected for histological observation, biochemical examination, and transcriptomic, metabolomic, and intestinal microbiome analysis. Our results showed that excessive ammonia nitrogen blocked the normal immune function and compromised the integrity of liver and gill tissues through oxidative stress-mediated differential cell death pathways. Meanwhile, the multi-omics analysis revealed that ammonia exposure predominantly altered the carbohydrate, lipid, and amino acid metabolism modes. In addition, it was also demonstrated that ammonia nitrogen stress affected the composition of intestinal microbiota taxa. This study provides insights into the potential risks and hazards of ammonia stress on cold fish in natural waters and provides a reference for the environment control of the water quality in aquaculture.

## 1. Introduction

In aquatic ecosystems, ammonia is regarded as one of the key environmental pollutants. It enters these environments from various sources, including industrial waste, sewage, biological waste decomposition, and agricultural run-off, causing harmful toxic effects to aquatic organisms [1,2]. Fish, as one of the most important components in aquatic environments, are susceptible to ammonia stress [3]. As such, the health of fish species has gained increasing attention. Ammonia is highly fat-soluble, readily entering the body through the gills, liver, and intestine [4]. Ammonia has been confirmed to be toxic to most aquatic animals, such as yellow catfish (*Pelteobagrus fulvidraco*) and rainbow trout (*Oncorhynchus mykiss*) [5,6]. Increased amounts of ammonia in the water body affect the excretory process of organisms, leading to a continuous accumulation of ammonia within them. Ammonia stress can inhibit the growth performance of fish and cause serious tissue injury mediated by oxidative stress and immune imbalance injury [7].

The liver is of great significance in the maintenance of metabolic balance through the removal of toxins from the body [8] and is regarded as a key indicator of oxidative stress in organisms [9]. The gills of bony fish play a critical role in osmotic, acid–base, ion regulation, and waste excretion. In addition, they can absorb a series of toxicants, such as ammonia [10]. Many studies have indicated that acute ammonia stress can result in significant histopathological alterations and osmotic dysfunction in the gills [11]. Multi-omics approaches have been widely used to explore the possible mechanisms of the adverse effects of environmental pollutants on fish. For instance, multi-omics has revealed the effects of copper exposure on the liver of rainbow trout (*Oncorhynchus mykiss*) [12]. Additionally, Li et al. [13] elucidated the tolerance mechanisms in the gills of *Paramisgurnus dabryanus* to subchronic carbonate alkalinity exposure by using multi-omics analysis. However, the response mechanisms to ammonia exposure in the liver and gills of Amur grayling are ill-defined.

In fish, ammonia accumulation rapidly results in an increase in reactive oxygen species (ROS), which subsequently induces oxidative stress injury [14,15]. Lipid peroxidation and the subsequent formation of malondialdehyde (MDA) can occur when ROS production increases beyond normal levels in an organism [16]. Fish can activate antioxidant defense systems, including superoxide dismutase (SOD), glutathione peroxidase (GSH-Px), and catalase (CAT), in the clearance process of ROS [17].

The taxa of the gut microbiota comprise various functional species that benefit the host and are involved in the physiological regulation response of organisms under compensatory stress conditions [18]. Some investigations have reported that the gut microbes in fish significantly influence host immunity and defense mechanisms [19]. Studies have indicated that ammonia can disrupt the intestinal microbiota community of yellow catfish (*Pelteobagrus fulvidraco*) [20].

Amur grayling (*Thymallus grubii*) is considered to be an economically significant species of cold freshwater fish. However, its population has declined markedly in past decades because of environmental pollution and overfishing. As such, Amur grayling is listed as an endangered species in the China Red Data Book of Endangered Animals [21]. This decline has triggered attempts to enhance stocks through the development of captive culture techniques and the release of hatchery-reared Amur grayling. However, the concentration of ammonia in farms may have adverse effects on the development and health of Amur grayling due to the species exhibiting a heightened sensitivity to chemical contaminants [22]. So far, few studies have addressed in detail the toxicity effects of ammonia exposure on the physiological response of the respiratory/detoxification organs in *Thymallus grubii*. It is imperative to comprehend the toxicity of ammonia exposure so as to elucidate the physiological and tolerance thresholds of fish in both wild and aquaculture settings. Therefore, this study investigates the effects of ammonia toxicity on the antioxidant enzyme activity, immune indices, histology, and intestinal microbiota of Amur grayling. This study supplied the molecular mechanisms for ammonia toxicity, which provided valuable insights into the tolerance mechanisms of *Thymallus grubii* to ammonia stress. Moreover, the ammonia exposure concentrations used in this study can also be utilized to formulate policies for the biology conservation strategy of wild *Thymallus grubii* populations.

## 2. Materials and Methods

### 2.1. Animals

All experimental fish (body length: 17.7 ± 0.5 cm; body weight: 64.69 ± 5.64 g) were collected from the Huma River of the upper Amur River in China during autumn using angling and cast nets and then transported to an indoor culture facility (Harbin, China). All fish were habituated for 2 weeks prior to the formal experiment in a temperature-controlled recirculating water tank (80.5 cm × 48 cm × 39 cm) to remove transport stress. The tap water utilized was aerated for 5 h and changed twice daily; the water temperature was kept at 8 °C, and the pH value was in the range of 6.7 ± 0.12. In addition, dissolved oxygen (DO) was controlled between 6.0 and 7.8 mg/L; and the light cycle was set to 12 h light/12 h dark. The concentration of total ammonia was 0.023 mg/L. The utilization of fish in this research project was formally sanctioned by the EU’s legislation and guidelines pertaining to the care and welfare of animals (Directive 2010(63)EU) [23], and all animal experimental procedures were meticulously conducted in accordance with the guidelines stipulated by the Laboratory Animal Ethics Committee of the Research Institute of Fisheries of the Heilongjiang River (No. 20230915-001).

### 2.2. Acute Toxicity Test (96 h Lethal Concentration of 50%, LC50)

Stage 1: A 50% lethal concentration at 96 h (LC50) of total ammonia nitrogen (T-AN) experiment was conducted following the standard methods published by the USEPA [24] to determine the acute toxicity to ammonia. The initial step was conducting a pilot study with the objective of determining the approximate sensitivity range of Amur grayling to ammonia. The target concentrations tested were 20, 420, 820, 1120, and 1520 mg/L T-AN based on the LC50 studies on burbot (*Lota lota*), yellow catfish (*Pelteobagrus fulvidraco*), and rainbow trout (*Salmo gairdneri*) [5,25,26]. The Amur grayling (*Thymallus grubii*) (63.36 ± 5.32 g, N = 10 per tank) were held in 15 150.7 L (80.5 cm × 48 cm × 39 cm) temperature-controlled, recirculating water tanks (3 replicate tanks per concentration).

No feeding was performed during the experiment, and the water was renewed on a daily basis in a semistatic manner by replacing the medium with a fresh NH_4_Cl (analytically pure) solution (100 g/L) [27]. The ammonia nitrogen concentration in each tank was measured at six-hour intervals by Nesslerization, with NH_4_Cl used as a standard [12]. Any fish exhibiting signs of mortality were removed immediately upon detection, and the time of removal was meticulously recorded.

Stage 2: The Amur grayling (*Thymallus grubii*) (63.81 ± 3.46 g) were treated with a more precise range of ammonia concentrations (20, 220, 420, 620, and 820 mg/L T-AN). Using linear regression and substituting a 50% probability of animal mortality, a median lethal concentration at 96 h was obtained [20]. The second-phase trial was conducted in accordance with the same methodology as that employed in the initial-phase study.

### 2.3. Ammonia Exposure Experiment and Sample Collection

Based on the above pre-experiment, 90 healthy and uniformly sized (64.32 ± 4.68 g) *Thymallus grubii* were randomly divided into 9 tanks (150.7 L), during which no feed was given. The concentrations of T-AN in each treatment group were as follows: high-ammonia group (96 h LC50: 436.83 mg/L), low-ammonia group (10% of 96 h LC50: 43.683 mg/L), and the control group was not treated with an exogenous ammonia supplement. Three replicates were established for each experimental group. The control group and the low-ammonia group were housed with 20 individuals of *Thymallus grubii* per tank, and the high-ammonia group was housed with 40 individuals per tank. Five fish were randomly selected from each tank at each time point (24, 48, 72, and 96 h). Before measuring the body length and weight, fish were anesthetized using 0.02% MS-22. Subsequently, the liver, gill, and intestine contents were immediately separated and loaded in RNase-free centrifuge tubes for biochemical measurement and multi-omics analysis. The samples were immediately frozen in liquid nitrogen for 3h and stored at −80°C. Meanwhile, partial liver, gill, and intestine tissues were fixed in 10% formaldehyde for histopathological observation.

### 2.4. Histopathological Observation of the Liver, Gill and Intestine

Following a 24-h fixation period, the tissue samples were subjected to ethanol dehydration, hyalinization in xylene, paraffin embedding, and sectioning at a thickness of 5 μm using an HM 325 microtome (MICROM, Walldorf, Germany). Sections were stained with hematoxylin and eosin (HE). Slides were examined by light microscopy (Eclipse Ci-L, Nikon, Tokyo, Japan) and photographed (×200 and ×400). The detailed procedure can be found in a previous study [28].

### 2.5. Biochemical Analysis Measurement

The activities of CAT (JL-T0900), GSH-Px (JL-T0879), SOD (JL-T0781), AKP (JL-T0946), ACP (JL-T1094), MDA (JL-T0761), LZM (JL-T1062), ALT (JL-T0873), AST (JL-T0875), and Na^+^/K^+^-ATPase (JL-T0659) and the ammonia (JL-T0741) content were measured using specific kits purchased from Jianglai Co., Ltd. (Shanghai, China). Enzyme activities were measured according to the manufacturer’s instructions. Optical density (OD) values were measured using an absorbance microplate reader (SpectraMax Plus 384, Molecular Devices, California, USA) at 405 nm (AKP), 405 nm (ACP), 532 and 600 nm (MDA), 510 nm (CAT), 412 nm (GSH-Px), 450 nm (SOD), 530 nm (LZM), 520 nm (ALT), 520 nm (AST), 700 nm (Na^+^/K^+^-ATPase), and 630 nm (ammonia).

### 2.6. cDNA Library Construction and Transcriptome Sequencing

The detailed procedure can be found in Appendix A.

### 2.7. Quantification and Differentially Expressed Genes (DEGs) Analysis

The detailed procedure can be found in Appendix B [29].

### 2.8. Quantitative Real-Time PCR (qRT-PCR) Verification

The detailed procedure can be found in Appendix C [30] (Table S1).

### 2.9. Untargeted Metabolomics Analysis

The detailed procedure can be found in Appendix D.

### 2.10. 16S Ribosomal DNA Sequencing and Gut Microbiome Analysis

The detailed procedure can be found in Appendix E.

### 2.11. Immunohistochemistry Assay

Fixed tissue was sectioned and subsequently treated with xylene to remove the paraffin, followed by a gradient ethanol solution. Sections were placed in citrate buffer (pH = 6.0) at 28 °C for antigen retrieval and then incubated with 3% H_2_O_2_ for 25 min to inactivate endogenous peroxidase. Following a blocking period of 65 min, sections were sealed in a desiccating oven after treatment with 3% bovine serum albumin (BSA). Primary antibodies (GCL, GPX-4, ferritin, HO-1, TNF-α, FAS, FASL, and Caspase 3) were added to the sections for incubation at 4 °C overnight, and then sections were incubated with horseradish peroxidase (HRP)-conjugated IgG for 70 min. A fresh diaminobenzidene (DAB) solution for color development was subsequently applied, after which the sections were counterstained with hematoxylin, dehydrated, and mounted. CaseViewer 2.4.0.119028 (3DHISTECH, Budapest, Hungary) was utilized to scan the sections. In the scanned images, the nuclei were shown in blue, while the positive expression regions of the target proteins appeared in brown.

### 2.12. Statistical Analysis

The biochemical data were calculated using Microsoft Excel and analyzed with the statistical software package SPSS 22.0. All experiments were performed with triplicate samples and repeated at least three times, and the data are presented as the mean ± standard deviation (SD). Prior to undertaking statistical analysis, the normality and the homogeneity of the variance of the data were ascertained via the Kolmogorov–Smirnov and Levene’s tests, respectively. The interactive effects of the ammonia treatments and stress time were analyzed via a two-way analysis of variance (ANOVA) with a least significance difference post hoc test. A value of *p* ≤ 0.05 indicated a significant difference between two groups. Finally, Image J V1.8.0 was utilized for the morphological measurements (villus height, villus width, muscular thickness, lamina propria width, and submucosal thickness).

## 3. Results

### 3.1. Acute Toxicity (96 h LC50) Experiments

In stage 1, the initial results indicated that the fish used in the experiment (body weight: 63.36 ± 5.32 g) did not experience mortality within the following 24 h when exposed to 20 mg/L T-AN. Furthermore, fish exposed to T-AN concentrations of 820, 1120, and 1520 mg/L exhibited mortality before 96 h. Therefore, the 96 h no-lethal maximum concentration (20 mg/L) and the 96 h all-lethal minimum concentration (820 mg/L T-AN) were set as the initial and the final concentrations of the altered range experiment, respectively.

In stage 2, the fish (63.81 ± 3.46 g) were exposed to T-AN target concentrations of 20, 220, 420, 620, and 820 mg/L for 96 h, respectively, and the results showed that mortality was dependent on the concentration of ammonia. The respective percentages were 0%, 25%, 50%, 66.7%, and 100% (Table 1). The estimated 96 h LC50 was calculated using the linear interpolation method (y = 0.0012x − 0.0242, R^2^ = 0.9918, where y = % of mortality and x = ammonia concentration), resulting in a value of 436.83 mg/L T-AN. Accordingly, the ammonia level was identified as the sole factor influencing mortality in this experiment.

### 3.2. Histopathology Observation for Gill, Liver and Intestine Tissues

**Figure 1:**
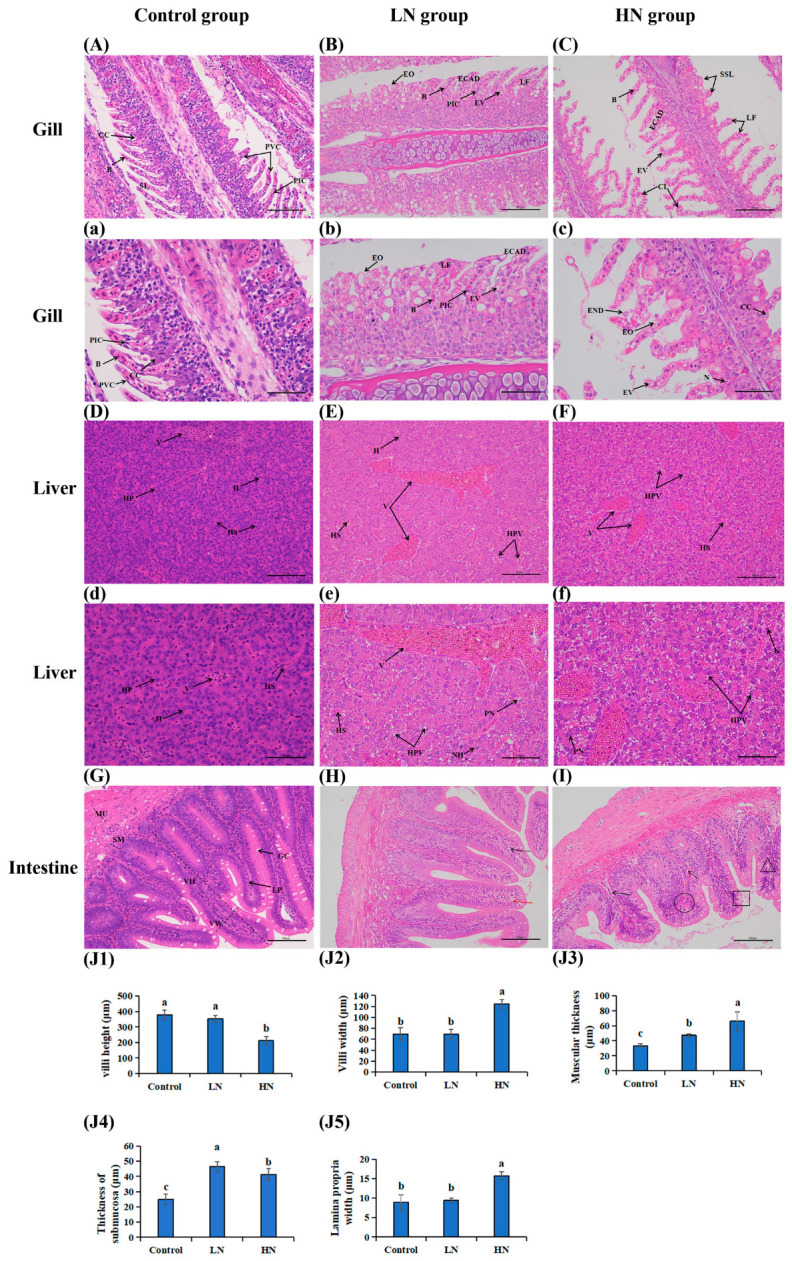
Gill, liver and mid-intestine histology of Amur grayling after stress to different concentration of ammonia for 96 h (HE). (**A**–**C**) represent different gill tissues (×200, scale bar=100 μm). (**a–c**) (×400, scale bar=50 μm). (**D**–**F**) represent different liver tissues (×200, scale bar=100 μm). (**d**–**f**) (×400, scale bar=50 μm). (**G**–**I**) represent different intestine tissues (×200, scale bar=100 μm); (**H**) the intestinal lamina propria (black arrows) and pillar cells vacuolation (red arrows); (**I**) lamina propria vacuolation (black arrows), cell arrangement disorder (black round), striate border damage (black rectangular) and cell death (black triangle). (**J1**–**J5**) Morphological measurements of villi height, villi width, muscular thickness, thickness of submucosa, and lamina propria width, respectively (mean ± SD, n = 7). Different small letters above the bars represents the statistical significance between each two groups. CC: chloride cells; B: blood cells; PIC: pillar cells; PVC: pavement cells; SL: secondary lamellae; EO: epithelial oedema; EV: cellular vacuolation; LF: lamellae fusion; ECAD: epithelial cells arranged disorder; SSL: shortening of secondary lamellae; CL: curling of secondary lamellae; END: epithelial necrosis and desquamation; N: nucleolysis; V: vascular; H: hepatocytes; HS: hepatic sinuses; HP: hepatic plate; HPV: hepato cellular vacuolation; NH: nuclear hypertrophy; PN: cellular peripheral nucleus; MU: muscularis; SM: submucosa; LP: lamina propria; GC: goblet cell; VW: villi width; VH: villi height.

In the control group (Figure 1A,a), the gill tissue displayed regular morphological structures in the pavement cells and secondary gill lamellae, with the pillar/pavement cells being well ordered without evident abnormalities. The gill tissue in the low-concentration group showed cellular vacuolation, lamellar fusion, and epithelial edema, with epithelial cells arranged in a disordered fashion (Figure 1B,b). The high-concentration samples exhibited severe curling of the secondary lamellae, secondary lamellar shortening, and epithelial cell necrosis, desquamation, and nucleolysis (Figure 1C,c).

With regard to the liver tissue, the hepatic sinuses, vacuoles, and hepatocytes were clearly observed in the control group (Figure 1D,d), with the hepatocytes being nearly polyhedric in shape and their nuclei being regularly distributed in the center of cells. The hepatic plate in the low-concentration group was distributed chaotically, with clear cellular peripheral nuclei and nuclear hypertrophy observed (Figure 1E,e). In the high-concentration group, hepatocyte damage was severe; the cell arrangement was extremely chaotic, more so than in the low-concentration group, and nuclei began to exhibit clear karyolysis (Figure 1F,f). Compared to the low-concentration group, the number of vacuoles in liver tissue was increased in the high-concentration group.

The villi were observed to be neatly arranged in the control group, and the epithelial cells were unbroken (Figure 1G). On the contrary, there was marked intestinal muscular and submucosal dilatation in the low-concentration group (LN group) (Figure 1H,J3,4), and the intestinal villi of fish were more swollen in the high-concentration group (HN group) (Figure 1I,J2) while displaying markedly increased muscular thickness (Figure 1I,J3) and lamina propria thickening (Figure 1I,J5). Meanwhile, the results demonstrated that exposure to varying concentrations of ammonia resulted in the formation of lesions of disparate types in the intestine of Amur grayling. Low concentrations induced reversible alterations, such as pillar cell vacuolation and lamina propria, while high ammonia concentration group mainly led to sparse mucous cells, cell arrangement disorder, striate border damage, and cell death.

### 3.3. Physiological and Biochemical Indices in Liver and Gill

**Figure 2:**
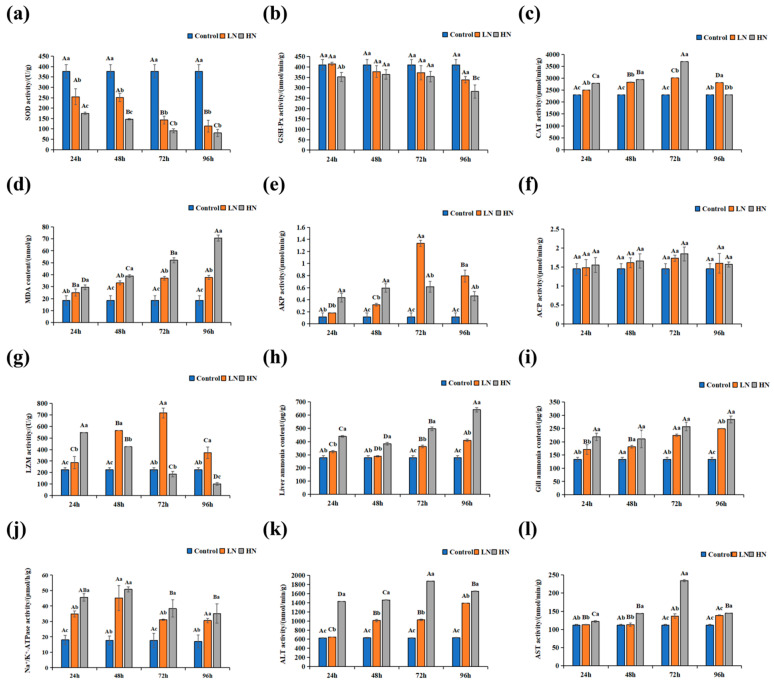
Effects of acute ammonia stress on liver and gill metabolic enzymes in adult Amur grayling (*Thymallus grubii*). (**a**) liver SOD; (**b**) liver GSH-Px; (**c**) liver CAT; (**d**) liver MDA; (**e**) liver AKP; (**f**) liver ACP; (**g**) liver LZM; (**h**) liver ammonia content; (**i**) gill ammonia content; (**j**) gill Na^+^/K^+^-ATPase; (**k**) liver ALT; (**l**) liver AST. Different capital letters above the bars indicate significant differences (*p* < 0.05) at different time points of same group and different small letters above the bars indicate significant differences (*p* < 0.05) in different groups at the same time point.

The oxidative stress and immunity-related indicator levels under ammonia exposure are displayed in Figure 2. SOD and GSH-Px activities decreased with an elevation in ammonia concentration at each designated time point. SOD activity in the ammonia-stress groups was reduced with increased exposure time, and SOD activity in the low- and high-ammonia groups after 72 h of exposure was markedly lower than that after 24 h/48 h (*p* ≤ 0.05) (Figure 2a). Similarly, GSH-Px activity declined with an increasing exposure time, but GSH-Px activity in the high-ammonia group after 96 h of exposure was significantly lower than that after 24 h, 48 h, and 72 h (*p* ≤ 0.05); its activity in the low-ammonia group was not significantly different with the rising exposure time (*p* > 0.05) (Figure 2b). CAT activity significantly increased with prolonged exposure concentrations at 24 h, 48 h, and 72 h (*p* ≤ 0.05), first increasing followed by a decrease at 96 h (Figure 2c). The MDA level markedly rose with rising ammonia concentrations at each time point (Figure 2d). The MDA level in the high-ammonia group markedly added with exposure time (*p* ≤ 0.05), and in the low-ammonia group, it was significantly higher after 48 h of exposure than after 24 h (*p* ≤ 0.05).

ACP activity was raised with increasing exposure concentrations at 24 h, 48 h, and 72 h (*p* > 0.05), first increasing and then declining at 96 h (Figure 2e). Similarly, the AKP activity was markedly increased at 24 h and 48 h with prolonged exposure concentrations (*p* ≤ 0.05). The AKP activity first increased and then reduced after 72 h with increasing exposure concentrations (Figure 2f). The activity of LZM in fish liver displayed a significant reducing trend in the high-ammonia concentration group (*p* ≤ 0.05); the low-ammonia group initially showed a significant increasing trend before decreasing (*p* ≤ 0.05), reaching a high at 72 h (Figure 2g).

The ammonia accumulation level and ALT, AST, and Na^+^/K^+^-ATPase activities in the liver of Amur grayling after 24, 48, 72, and 96 h are shown in Figure 2h–l. The ammonia content in liver and gill tissues rose with rising ammonia concentrations at each time point (Figure 2h,i). Moreover, the ammonia content in the liver of each concentration group first significantly declined and then increased when the exposure time increased (*p* ≤ 0.05). ALT, AST, and Na^+^/K^+^-ATPase activities also increased with increasing ammonia concentrations at each time point. ALT and AST activities in the low-ammonia group demonstrated elevation with exposure time, while ALT and AST activities in the high-concentration group were markedly added before being inhibited by an increased exposure time (*p* ≤ 0.05) (Figure 2j,k). Na^+^/K^+^-ATPase activity first increased and then reduced with the exposure duration (Figure 2l).

### 3.4. Transcriptome Profiling and Annotation

In order to explore the molecular response mechanisms of *Thymallus grubii* to ammonia stress, liver and gill tissues from the ammonia-stress and control groups were collected for the reference genome-free transcriptomic analysis. A total of 419, 458, and 119 clean reads were obtained from the 18 samples based on transcriptomic sequencing, and the average GC content, Q20, and Q30 values in the clean data were 46.4%, 97.85%, and 94.24%, respectively (Table S2).

### 3.5. DEGs Analysis

**Figure 3:**
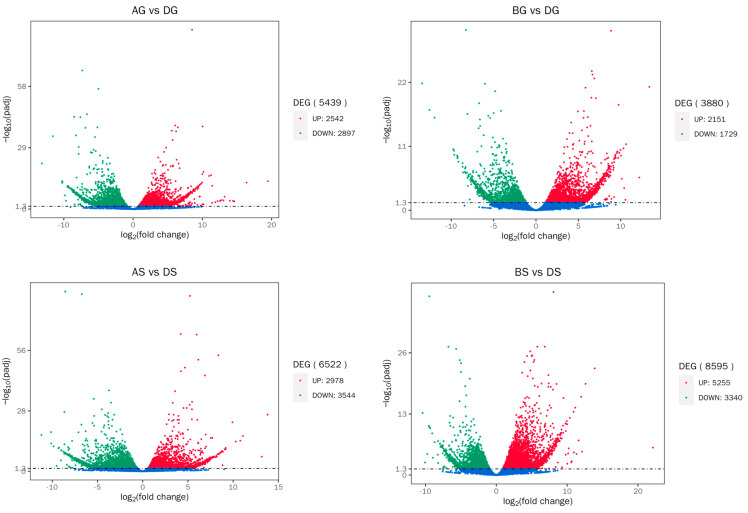
Volcano plot of DEG distribution trends in liver (AG vs. DG, BG vs. DG) and gill ( AS vs. DS, BS vs. DS). Each dot represents one gene. Blue dots represent non-differentially expressed genes. G: liver; S: gill; AG and AS: low-ammonia exposure group; BG and BS: high-ammonia exposure group; DG and DS: control group.

Compared to the DG group, a total of 5439 (2542 upregulated and 2897 downregulated) and 3880 (2151 upregulated and 1729 downregulated) DEGs were detected in the AG and BG groups, respectively. Compared to the DS group, a total of 6522 (2978 upregulated and 3544 downregulated) and 8595 (5255 upregulated and 3340 downregulated) DEGs were marked in the AS and BS groups, respectively (Figure 3). Specifically, ferroptosis-related (hmox, fth1, gcl, gpx4), immune-related (tnfa, dusp), energy-metabolism-related (suclg2, aco2, ogdh, cs), and ammonia-detoxification-related (hk, gck, fbp1b, pgm1, pfkma) genes were identified (Table 2).

### 3.6. GO and KEGG Enrichment Analysis for DEGs

**Figure 4:**
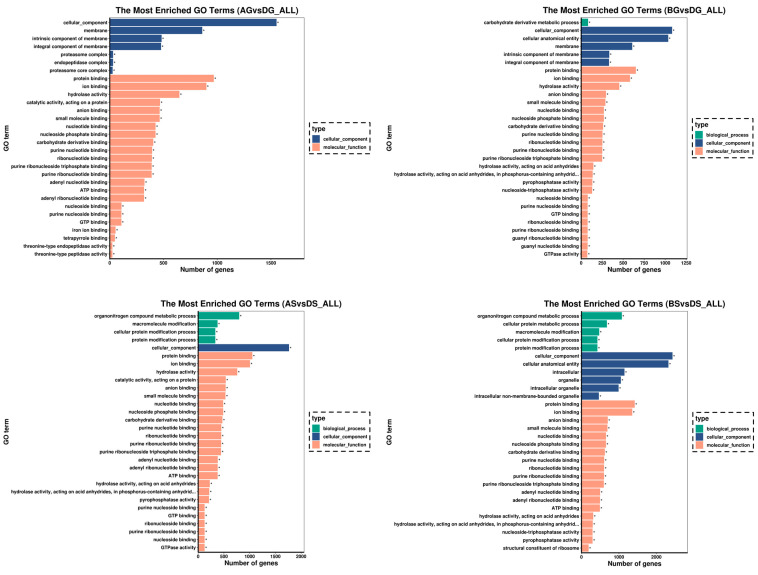
Bar chart showing the significantly enriched GO terms in liver and gill. Different colors represent the enriched GO categories. * indicate significant differences (*p* < 0.05).

In the liver, the most represented GO term that was annotated in the biological process was the carbohydrate derivative metabolic process; the most represented GO terms annotated in the molecular function were protein binding, ion binding, and hydrolase activity; and the most represented GO terms annotated in the cellular component were cellular component, cellular anatomical entity, and membrane (Figure 4). For the gills, the most represented GO terms annotated in the biological process was the organonitrogen compound metabolic process; the most typical GO terms annotated in the molecular function were involved in protein binding, ion binding, and hydrolase activity; and the most classical GO terms annotated in the cellular component were cellular component, cellular anatomical entity, and intracellular.

**Figure 5:**
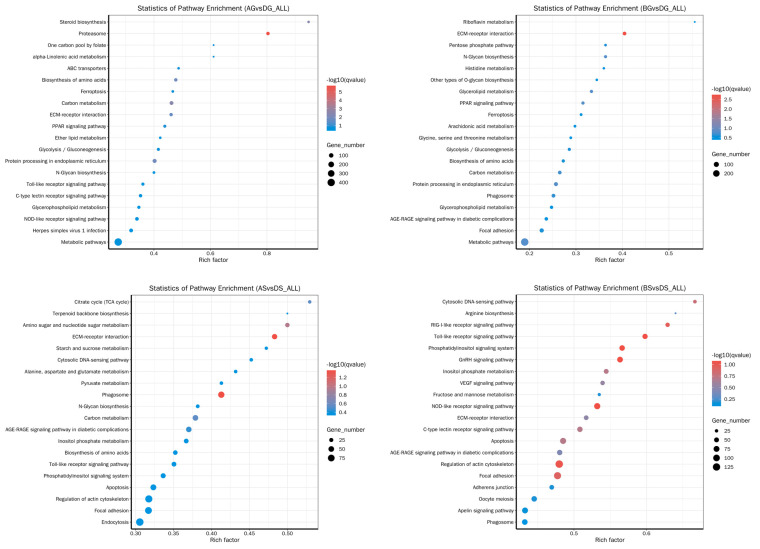
Bubble map showing the top 20 enriched KEGG pathways of DEGs in liver.

In the comparison between the control and test groups of the liver, the identified differentially expressed genes were predominantly associated with carbon metabolism (dre01200), glycolysis/gluconeogenesis (dre04216), proteasome (dre03050), and the biosynthesis of amino acids (dre01230), which are involved in carbohydrate and protein metabolism and related to amino acid metabolism involved in ammonia detoxification. Additionally, the immune function- and cell death-related signaling pathways (e.g., C-type lectin receptor signaling pathway (dre04625), ferroptosis (dre04216), phagosome (dre04145)) were enriched (Figure 5). In the comparison between the control and test groups of the gills, the most highly enriched pathways were related to amino acid metabolism, which plays a role in ammonia detoxification, either directly or indirectly: pyruvate metabolism (dre00620), arginine biosynthesis (dre00220), amino sugar and nucleotide sugar metabolism (dre00520), and the biosynthesis of amino acids (dre01230). In the comparisons of AS vs. DS and BS vs. DS, carbohydrate and lipid metabolism pathways, such as the citrate cycle (TCA cycle) (dre00020), starch and sucrose metabolism (dre00500), carbon metabolism (dre01200), and inositol phosphate metabolism (dre00562), were significantly enriched. Furthermore, immune function- and cell death-related signaling pathways (e.g., Toll-like receptor signaling pathway (dre04620), focal adhesion (dre04510), phagosome (dre04145), endocytosis (dre04144), C-type lectin receptor signaling pathway (dre04625), apoptosis (dre04210)) were also enriched. Additionally, the KEGG enrichment analysis revealed that the “MAPK” pathway was enriched in the BS vs. DS comparison.

### 3.7. Changes in Metabolite Levels in Fish Live and Gills

**Figure 6:**
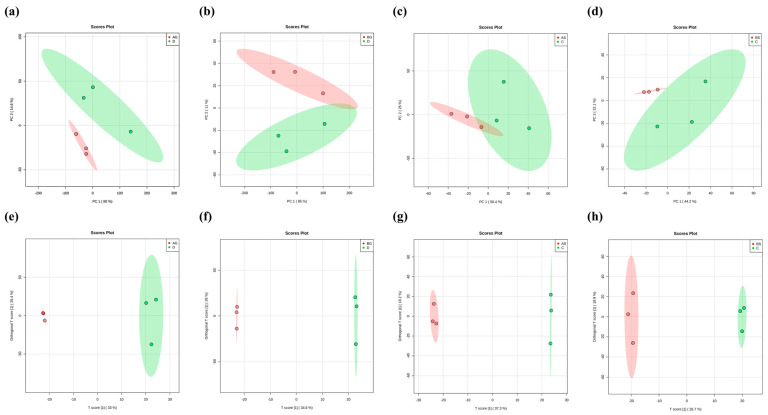
(**a**–**d**) PCA of LC-MS metabolite profiles of four comparison groups. (**e**–**h**) OPLS-DA of LC-MS metabolite profiles of four comparison groups. Notes: AG: low-ammonia concentration group of liver; D: control group of liver, BG: high-ammonia concentration group of liver, AS: low-ammonia concentration group of gill C: control group of gill, BS: high-ammonia concentration group of gill.

**Figure 7:**
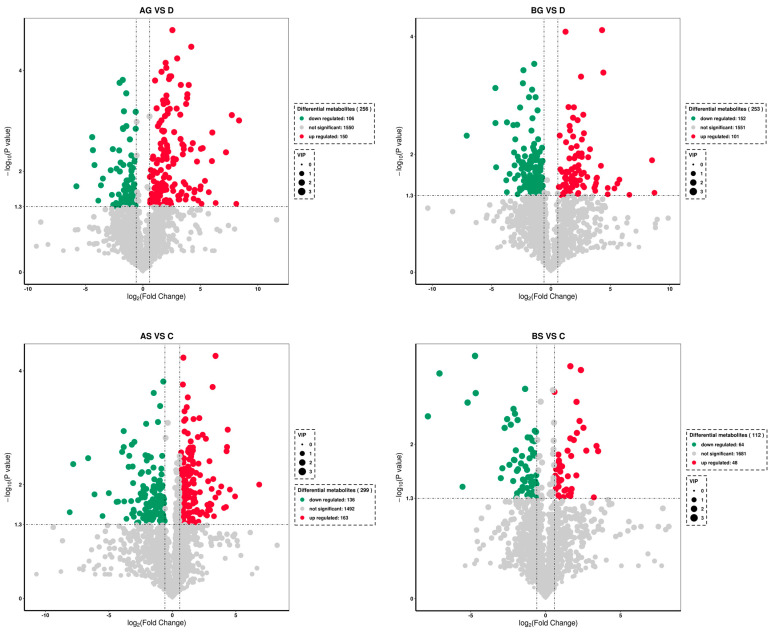
Volcano plots of differential metabolites in four comparison groups.

**Figure 8:**
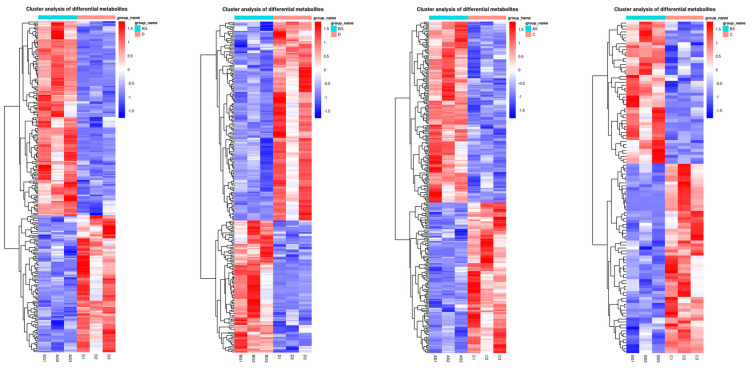
The clustering heatmap showing the differential metabolites in liver and gill.

**Figure 9:**
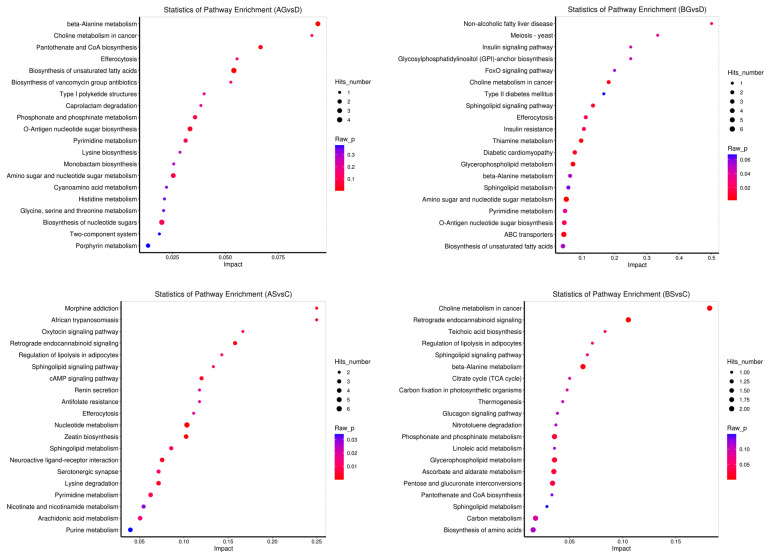
Bubble plots of the top 20 pathways enriched by KEGG of differential metabolites in the four comparison groups.

The metabolomic analysis showed that ammonia exposure resulted in various differentially expressed metabolites (DEMs) in fish liver and gills. The PCA (Figure 6a–d) and OPLS-DA (Figure 6e–h) results showed that the expression levels of some metabolites varied significantly between the control and ammonia-stress groups (*p* ≤ 0.05). In total, 256, 253, 299, and 112 DEMs were identified in the AG vs. D, BG vs. D, AS vs. C, and BS vs. C comparisons, respectively (Figure 7). Most of these metabolites were classified as “carbohydrates and carbohydrate conjugates”, “glycerophosphocholines”, and “amino acids, peptides, and analogues” (Figure 8). Further study identified the top 20 significantly different metabolite pathways in the KEGG analysis (Figure 9). In the liver, the metabolites were significantly enriched in pyrimidine metabolism, beta-alanine metabolism, lysine biosynthesis, glycine, serine and threonine metabolism, and biosynthesis of unsaturated fatty acids. In the gills, the metabolites were significantly enriched in lysine degradation, bate-alanine metabolism, TCA cycle, carbon metabolism, and the biosynthesis of amino acids.

### 3.8. Intestinal Microbe Composition of Grouper After Stress

**Figure 10:**
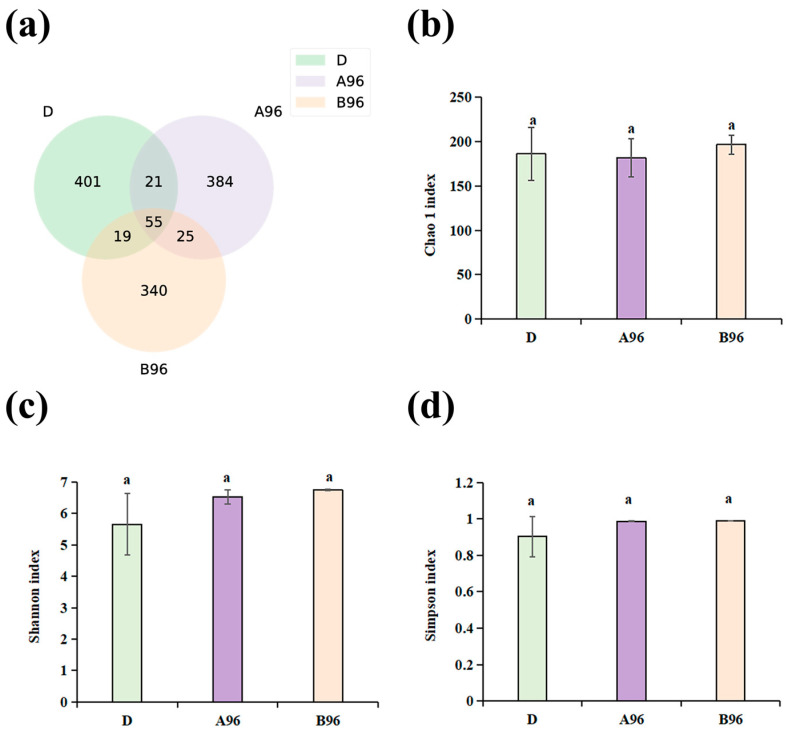
(**a**) Venn diagram of shared and specific ASVs among groups. Community richness index including (**b**) Chao1; Community diversity index including (**c**) Shannon and (**d**) Simpson. The presence of the same small letters above the bars indicates that there are no significant differences (*p* > 0.05) between the different groups.

Intestinal microbe sequencing was performed for the intestinal contents of the control (D), low-ammonia concentration (A96), and high-ammonia concentration (B96) groups after 96 h of exposure. We found a total of 723,138 raw reads from nine samples of *Thymallus grubii*. A Venn diagram indicated that 55 amplicon sequence variants (ASVs) were present in the three groups, and the number of unique ASVs was 401, 384, and 340 in the D, A96, and B96 groups, respectively (Figure 10a). Ammonia exposure reduced the gut microbiota; the higher the concentration, the lower the ASVs. To test whether the gut microbiota diversity varied relative to different ammonia concentrations, the Chao1, Shannon, and Simpson indices were utilized to calculate the alpha diversity. The results showed that the Chao1 index in the low-ammonia group was the lowest (Figure 10b), whereas the Shannon and Simpson indices in the control group were the lowest (Figure 10c,d). The three indices presented no significant differences between the control and treatment groups.

**Figure 11:**
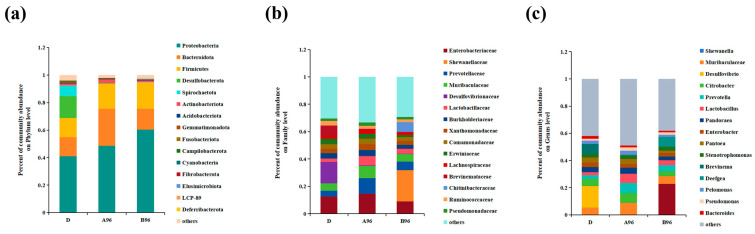
(**a**–**c**) Community bar plot analysis at phylum, family, and genus level.

In the control group, the intestinal bacterial abundance showed that the predominant phylum was Proteobacteria (40.93%), followed by Bacteroidota (13.95%), Firmicutes (13.94%), Desulfobacterota (15.78%), Spirochaetota (7.16%), and Actinobacteriota (1.33%). However, in the ammonia-exposed groups (A96 and B96), Proteobacteria increased by 7.67% and 19.36%, respectively, Bacteroidota increased by 12.97% and 1.27%, respectively, and Firmicutes increased by 4.35% and 5.09%, respectively, while Desulfobacterota decreased by 15.51% and 15.6%, and Spirochaetota decreased markedly by 7.136% and 7.138%, respectively. Similarly, Actinobacteriota greatly increased by 1.14% in the A96 group and decreased by 0.13% in the B96 group compared to the control group (Figure 11a). At the family level, *Enterobacteriaceae*, *Prevotellaceae*, *Muribaculaceae*, and *Lactobacillaceae* occupied the dominant positions after ammonia stress. *Prevotellaceae*, *Muribaculaceae*, *Lactobacillaceae*, *Xanthomonadaceae*, and *Pseudomonadaceae* were more abundant in the ammonia-exposed groups (A96 and B96) than the control, while *Desulfovibrionaceae*, *Erwiniaceae*, *Brevinemataceae*, and *Ruminococcaceae* were lower in the control group. *Enterobacteriaceae*, *Burkholderiaceae*, *Comamonadaceae*, and *Lachnospiraceae* decreased with increasing ammonia concentrations (Figure 11b). At the genus level, *Muribaculaceae*, *Citrobacter*, *Prevotella*, and *Lactobacillus* occupied the dominant positions after ammonia stress. The abundance of *Muribaculaceae*, *Prevotella*, *Lactobacillus*, *Stenotrophomonas*, and *Pseudomonas* in the ammonia-exposed groups (A96 and B96) was increased compared to the control, while *Desulfovibrio*, *Enterobacter*, *Pantoea*, *Brevinema*, and *Bacteroides* were lower in the control group. *Citrobacter*, *Pandoraea*, and *Pelomonas* decreased with increasing ammonia concentrations (Figure 11c).

**Figure 12:**
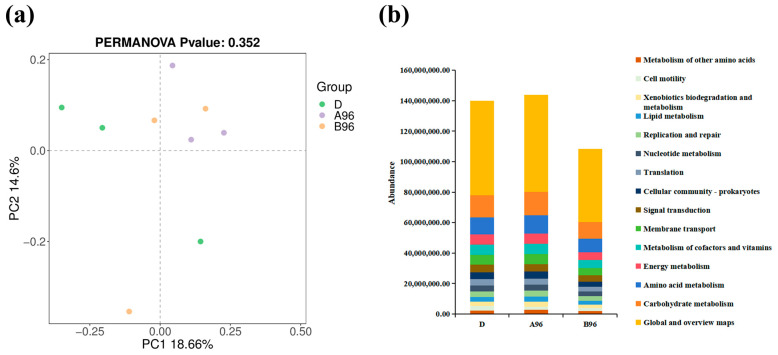
(**a**) PCoA analysis of community difference of three treatment. (**b**) KEGG pathway abundance statistics analysis after exposure to ammonia for 96 h.

The PCoA illustrated that samples did not cluster at any ammonia concentration point (Figure 12a). A functional prediction of the intestinal microbiota was identified, comprising the top 15 functional categories (Figure 12b). The KEGG pathway abundance analysis revealed a reduction in the abundance of fifteen functional categories in the high-ammonia group, including energy metabolism of other amino acids, and carbohydrate metabolism.

### 3.9. Validation for RNA-Seq Profiling

To verify the credibility of the transcriptome sequencing results, ten DEGs were randomly chosen from the AG vs. DG (mmp17 and vtg), BG vs. DG (mmp17, lama1, nfm, and tip41), AS vs. DS (shop21 and gimap7), and BS vs. DS (nin and adipor2) comparisons (Figure S1). It was shown that the trends of the changes in these candidate genes were in agreement with the results provided by the transcriptome project.

### 3.10. Differential Cell Death Pathways Induced by Low/High Concentration Ammonia Stress

**Figure 13:**
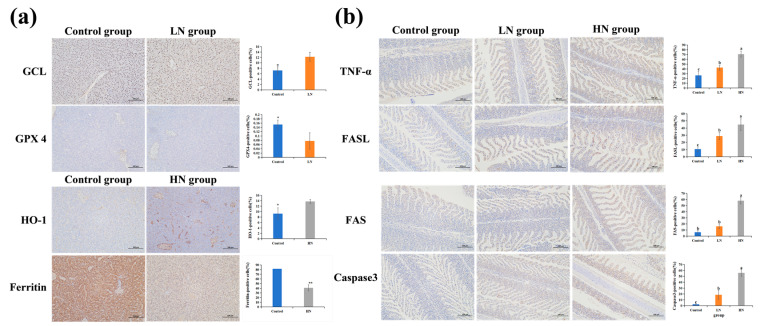
Immunohistochemical staining for the ferroptosis (**a**) and apoptosis (**b**)-related proteins in Amur grayling liver and gill tissues (200×). Scale bar, 100 μm (n = 3). Quantifications of IHC staining are shown in the panel right. The nucleus was observed to be blue, while the positive expression area of the target protein was identified as brown. * above the bars represents significant differences (*p* ≤ 0.05) between groups. ** above the bars represents significant differences (*p* < 0.01) between groups. Different small letters above the bars indicate significant differences (*p* < 0.05) in different groups.

Immunohistochemistry was employed to examine the protein expression levels of apoptosis-related biomarkers (TNF-α, glutamate cysteine ligase (GCL), factor-related apoptosis (FAS), factor-related apoptosis ligand (FASL), and Caspase 3) and ferroptosis regulators (ferritin, heme oxygenase-1 (HO-1), and glutathione peroxidase (GPX4)) (Figure 13). In the liver, compared to the control group, significantly higher HO-1 and ferritin levels were found in the high-ammonia stress group (*p* ≤ 0.05). Similarly, a significantly higher GCL level was found in the low-ammonia stress group, while GPX4 protein expression was reduced significantly (*p* ≤ 0.05). In the gills, TNF-α, FASL, FAS, and Caspase 3 protein levels were significantly increased with prolonged exposure concentrations (*p* ≤ 0.05).

## 4. Discussion

Ammonia is one of the main harmful pollutants in water environments, possessing a physiological irritability to aquatic animals. Ammonia is capable of readily permeating the majority of biological membranes, resulting in a significant impairment of various organ systems [31]. This study has shown that low/high concentrations of ammonia exposure can markedly grow the ammonia content in the liver and gills, suggesting that the ammonia present in the external environment is ingested and subsequently stored in those organs. The results obtained in the present study are in line with those previously reported in similar studies that have shown that ammonia can accumulate in the tissues of zebrafish (*Danio rerio*) and yellow catfish under ammonia stress [20,32].

Ammonia exposure can induce the production of ROS [33]. It has been suggested that the antioxidant defense system of fish prevents ROS production by activating antioxidant enzymes [34]. Antioxidant enzymes, such as CAT, SOD, and GSH-Px, help maintain the balance of the antioxidant system under oxidative stress, thus safeguarding cells from oxidative damage. SOD converts superoxide anions to H_2_O_2_, which is then converted by CAT to H_2_O [35]. GSH-Px is a widely distributed enzyme that catalyzes the decomposition of H_2_O_2_, thereby protecting cell membrane integrity and functionality [36]. MDA is typically employed as a biomarker of lipid peroxidation, given its status as a pivotal product of membrane lipid peroxidation in the wake of a free radical assault on biological membranes [37]. In this experiment, the activities of CAT increased in the initial stage of stress, which the oxidation system initiated in response to the ammonia stress. Meanwhile, SOD and GSH-Px significantly reduced after stress, and the activities of CAT decreased at 96 h. This evidence demonstrates that antioxidant enzyme activity is suppressed in response to ammonia stress. As a result, the antioxidant capacity of Amur grayling decreased, and their natural mechanisms for removing oxygen free radicals produced over time were found to be limited. It is possible that this may have resulted in a disruption to the equilibrium between the antioxidant system and oxidative stress in Amur grayling, which could subsequently lead to a loss of compensatory mechanisms due to excessive ROS. This resulted in an acceleration of lipid peroxidation and an increase in MDA, due to the involvement of oxygen free radicals. This finding aligns with the outcomes of other studies investigating the impact of stress [38].

The liver plays a crucial role in the body’s defense mechanisms. It has several enzymes involved in immunity, such as AKP, ACP, and LZM, which are of particular importance for sustaining health and providing protection against foreign substances [39,40]. The activities of AKP and ACP in yellowfin tuna (*Thunnus albacares*) demonstrated an initial increase during the early stages of ammonia stress, followed by a subsequent decline during the later stages [41]. In this study, the AKP and ACP in Amur grayling exposed to low and high ammonia concentrations were observed to be elevated at 24, 48, and 72 h with increasing concentrations. However, these enzyme activities demonstrated a declining trend at 96 h, which is consistent with the outcomes previously documented. The activities of LZM demonstrated an increasing trend before 72 h in the low-ammonia group but reduced after 96 h, while they exhibited an upward trend with increasing time in the high-ammonia group. The findings indicate that the immune capacity of adult Amur grayling may initially be enhanced by ammonia stress. However, as the stress duration increases, the immune system’s resilience may diminish, leading to a rapid reduction in enzyme activity and an imbalance in the immune system. Similar results have also been observed in juvenile turbot (*Scophthalmus maximus*) [42].

The present study demonstrated that one of the principal pathways that exhibited a marked reduction in expression and may exert a substantial influence on growth performance is the mitogen-activated protein kinase (MAPK) signaling pathway. The MAPK signaling pathway was significantly downregulated in the gills of Amur grayling exposed to a high concentration of ammonia. MAPK activity is controlled by a group of phosphatases called MAPK phosphatases (MKPs) that dephosphorylate MAPKs, causing their inactivation [43]. Kültz and Kristina [44] showed a decrease in MAPK activity in the gills of euryhaline fish exposed to osmotic stress. Our study demonstrated that the expression of the MKP-related gene (dusp) was upregulated with a high concentration of ammonia exposure in the gills. Huang et al. [45] demonstrated that inhibiting MAKP activation induced the cell apoptosis process. All Toll-like receptor signaling pathways ultimately cause activation of the transcription factor nuclear factor kappa B (NF-kB), which regulates the expression of the TNF-α gene [46]. TNF-α is an important member of the TNF cytokine family that induces cell proliferation, inflammation, and general immune system stimulation [47]. Based on the DEG analysis provided by the transcriptome results, our study found that ammonia stress could induce the upregulation of inflammatory cytokine genes (tnfa), which is in agreement with similar results observed in puffer fish (*Takifugu obscurus*) [11].

Our study showed that the ferroptosis/apoptosis-related signaling pathway was enriched in the liver and gill tissues of Amur grayling under ammonia stress, respectively. It might suggest that the organism activates different cell death modes to resist the external physiological stress response caused by ammonia exposure. Interestingly, the transcriptome results clarified that low/high concentrations of ammonia exposure resulted in disparate mechanisms of ferroptosis in the liver of Amur grayling. A high concentration of ammonia led to cell ferroptosis by activating the HO-1/ferritin signaling pathway, while a low dose of exposure led to ferroptosis via activating the GCL/GPX4 signaling pathway. Various studies have shown that HO-1 plays a critical role in the ferroptosis process [48]. Ferritin is the major intracellular iron storage protein complex, which processes ferroxidase activity to oxidize Fe2+ to Fe3+ [49]. The upregulation of HO-1 enhances the degradation of heme, thereby leading to Fe2+ overload [50]. Iron overload can promote ROS generation to induce the peroxidation reaction and oxidative stress damage, which eventually mediates the ferroptosis process to clear damaged cells [51]. GPX4 is able to transform lipid hydrogen peroxide into non-toxic lipid alcohol, which protect cells from lipid peroxidation. Studies have shown that both HO-1 upregulation and GPX-4 downregulation can directly trigger the ferroptosis process mediated by excessive ROS production [52]. It is well established that both FASL/FAS signaling and TNF-α signaling can induce apoptosis in a range of tissues [53,54]. FASL is mainly expressed in immune cells [55]. FASL-containing inflammatory cells may cause the induction of apoptosis through the interaction with FAS-bearing target cells [56]. Caspase is one main family of cysteine proteases that plays an important role in both extrinsic and intrinsic pathways directly associated with programmed cell apoptosis [57]. Our study demonstrated that FAS, FASL, TNF-α, and Caspase 3 expression levels were markedly elevated in the gills after ammonia exposure. In addition, higher HO-1 protein and lower ferritin levels were found after high-dose ammonia stress, while a low concentration of ammonia exposure contributed to increased GCL expression and decreased GPX-4 expression. Therefore, these results suggest that Amur grayling activate tissue-specific cell-death pathways in their liver/gills in response to environmental ammonia stress and also initiate differential physiological mechanisms in hepatocytes to mediate active cell ferroptosis.

Prior studies have demonstrated that metabolic pathways, including lipid metabolism, TCA, and glycolysis, are the primary sources of energy for biological organisms. The maintenance of physiological processes in aquatic animals, including osmotic regulation, tissue injury repair, and ammonia excretion requires a greater input of energy in the presence of ammonia exposure [58]. In this study, a TCA-related metabolite (isocitric acid) was significantly inhibited at the high concentration of ammonia exposure, and many lipid-related metabolites (docosahexaenoic acid (DHA), docosapentaenoic acid (DPA), and eicosapentaenoic acid) were significantly activated. The expression of numerous TCA-associated genes (e.g., suclg2, cs, aco2 and ogdh) was markedly elevated, whereas the expression of many glycolysis-related genes (hk, gck, fbp1b, pgm1, and pfkma) was markedly reduced. These results show that ammonia stress impedes glycolysis while stimulating TCA and lipid metabolism, thereby providing Amur grayling with the energy resources necessary to cope with adversity.

Studies have demonstrated that glutamine and alanine synthesis and the inhibition of amino acid catabolism play a significant role in the detoxification of ammonia in aquatic animals by reducing ammonia production [59,60]. Glutamate binds to ammonia and synthesizes glutamine via GS when aquatic animals are in a high-ammonia environment [61]. The conversion of glutamate to alanine and aspartate is catalyzed by the enzymes ALT and AST, respectively, with the notable feature that ammonia is not produced during this process. The detoxification pathway of ammonia has been evidenced in both yellow catfish (*Pelteobagrus fulvidraco*) and rainbow trout (*Oncorhynchus mykiss*) [20,60]. Wang et al. [20] reported that ammonia stress can increase the amount of various amino acids. This study showed that the activities of AST and ALT in the liver were enhanced, and the expression of GS-related genes (glula, glulb, and glulc) was upregulated in the liver and gills after high-ammonia stress. In this study, KEGG analysis showed that the biosynthesis of amino acids pathway was upregulated during low-ammonia stress. These results show that the liver and gills can accomplish ammonia detoxification through amino acid metabolism, thereby decreasing the accumulation of ammonia in those organs. Na^+^/K^+^-ATPase plays an important role in the osmotic balance of fish, and excessive ammonia can modify the structure of Na^+^/K^+^-ATPase proteins to inhibit its enzyme activity. Na^+^/K^+^-ATPase is one protease that has the function of secreting organelle membranes and chloride cells in gill tissue. This enables the pumping of Na^+^ for K^+^ uptake, thereby maintaining normal osmotic pressure in the fish body. NH_4_^+^ can substitute K+ in the enzyme-binding site [62]. Therefore, Na^+^/K^+^-ATPase is tightly linked to the transport of NH_4_^+^ [63]. In this research, Na^+^/K^+^-ATPase activity increased markedly after 24 h of ammonia stress. Subsequently, after 72 h of ammonia stress, NNa^+^/K^+^-ATPase activity decreased; our results are similar to other research on *Eleutheronema tetradactylum* [64]. This indicates an early adaptive response of adult fish to ammonia stress, which may have increased Na^+^/K^+^-ATPase synthesis or added other pathways to improve Na^+^/K^+^-ATPase activity, thereby enhancing the ability to regulate ammonia concentrations in the body and reduce the associated negative effects. Na^+^/K^+^-ATPase activity decreased after 72 h of ammonia stress, with the reason being that ammonia leaches into the body from the environment, preventing its elimination from the body. The trend of a decrease in Na^+^/K^+^-ATPase activity could be attributed to the penetration of environmental ammonia into the organism. Ammonia infiltration counteracts the natural capacity of organisms to excrete ammonia, resulting in excessive ammonia accumulation in the body. In particular, it can modify the structure of Na^+^/K^+^-ATPase proteins, thereby decreasing their enzymatic activity.

The microbial communities within the gut microenvironment are of critical importance with regard to development, homeostasis, and protection against pathogens [65]. Additionally, the inflammatory response and oxidative stress are often linked to an imbalance in the intestinal microbiota [66]. Research has demonstrated that ammonia has the capacity to modify the composition of the intestinal microbiota in fish [67]. In the present study, after acute ammonia stress, the relative abundance of Proteobacteria, Bacteroidota, and Firmicutes increased. Proteobacteria are associated with the restoration of the carbon complex in fish, which is a sign of intestinal microbial disruption and inflammation [68,69]. Bacteroidota are involved in the decomposition of polymeric organic matter [70], while Firmicutes promote energy production through lipid metabolism [71]. Thus, Amur grayling may reduce inflammatory responses and oxidative stress by enhancing the abundance of intestinal dominant bacteria. The KEGG functional analysis also indicated that intestinal microorganisms were enriched in amino acid metabolism, carbohydrate metabolism, membrane transport, lipid metabolism, and energy metabolism, which may have caused the increasing abundances of Bacteroidota, Firmicutes, and Proteobacteria. When more energy is required to control osmotic balance and defend against pathogens, glucose metabolism happens through glycolysis or TCA under hormonal control. Therefore, many of the liver’s glycogen stores are exhausted, which may cause liver cells to swell and vacuolate [72]. In this study, the alpha and beta diversity did not differ significantly between groups, which may be due to individual factors in Amur grayling. Research findings have demonstrated that, in comparison with environmental influences, the fish itself exerts a significantly more substantial influence on its gut microbiome [73]. In addition, the gut microbiome of carnivores may be related to their own genes, and their alpha diversity is not markedly different [74]. Meanwhile, the composition of gut microbial communities from Atlantic salmon did not differ between the different environments [75]. Therefore, the results of this study provide direct evidence that the environment may not be the primary factor contributing to the structure of the gut microbial communities of Amur grayling.

## 5. Conclusions

To summarize, comprehensive analyses indicated that exposure to ammonia stress led to oxidative stress, inflammatory responses, energy metabolism imbalance, and apoptosis/ferroptosis in multiple organs of Amur grayling. This study highlighted the urgent need for controlling the ammonia level in water to ensure the welfare conditions of Amur grayling in aquaculture and natural habitats. Additionally, this study provided the potential critical ammonia concentration that would pose a threat to Amur grayling.

## Figures and Tables

**Table 1:** The mortality parameter was determined through two 96 h LC50 experiments on adult Amur grayling.

Target T-AN (mg/L)	Measured T-AN (mg/L)	Mortality (%)
Range finding experiment		
20	20.06 ± 0.08	0%
420	419.88 ± 0.22	50%
820	820.06 ± 0.09	100%
1120	1120.02 ± 0.13	100%
1520	1520.01 ± 0.1	100%
Modified range experiment		
20	20.04 ± 0.07 ^a^	0%
220	220.08 ± 0.38 ^b^	25%
420	419.83 ± 0.47 ^c^	50%
620	619.81 ± 0.40 ^d^	66.70%
820	819.81 ± 0.39 ^e^	100%

Notes: Measured T-AN values are presented as mean ± SD (n = 3). Different letters indicate significant differences (*p* < 0.05).

**Table 2:** The detailed description of DEGs and KEGG pathway associated with apoptosis, ferroptosis, immune, energy metabolism, and ammonia detoxification.

	Gene	Name	Log2FC	*p*-Value (×10^−5^)	Change	KEGG Pathway
AS vs. DS	*suclg2*	succinyl-CoA ligase	1.0355	88.689	up	Citrate cycle (TCA cycle)
	*cs*	citrate synthase	1.486	2.300	up	
	*aco2*	aconitate hydratase	0.90375	52.668	up	
	*ogdh*	2-oxoglutarate dehydrogenase-like	1.2881	117.650	up	
BS vs. DS	*suclg2*	succinyl-CoA ligase	2.1689	324.200	up	Citrate cycle (TCA cycle)
	*ogdh*	2-oxoglutarate dehydrogenase-like	2.4386	2.368	up	
	*dusp*	dual specificity protein phosphatase 3	1.4402	281.330	up	MAPK signaling pathway
	*tnfa*	tumor necrosis factor a	6.6264	13.803	up	Toll-like receptor signaling pathway
	*glul*	glutamate-ammonia ligase	1.4799	259.480	up	Arginine biosynthesis
AG vs. DG	*hk*	hexokinase	−2.4433	381.370	down	Glycolysis/Gluconeogenesis
	*gck*	glucokinase	−9.673	0.387	down	
	*fbp1b*	fructose-1,6-bisphosphatase	−1.9719	0.000	down	
	*pgm1*	phosphoglucomutase-1	−2.2693	20.512	down	
	*pfkma*	6-phosphofructokinase	−2.1265	11.893	down	
	*gcl*	glutamate--cysteine ligase	1.4309	98.206	up	Ferroptosis
	*gpx4*	glutathione peroxidase 4	−4.7869	0.000	down	
BG vs. DG	*hk*	hexokinase	−2.2626	100.890	down	Glycolysis/Gluconeogenesis
	*pgm1*	phosphoglucomutase-1	−2.4723	123.090	down	
	*pfkma*	6-phosphofructokinase	−2.2945	75.656	down	
	*hmox*	heme oxygenase 1	2.3756	18.129	up	Ferroptosis
	*fthl*	ferritin	4.6697	0.076	up	
	*glul*	glutamate-ammonia ligase	2.2101	80.732	up	Arginine biosynthesis

Notes: G: liver; S: gill; AG and AS: low-ammonia exposure group; BG and BS: high-ammonia exposure group; DG and DS: control group.

## Data Availability

The data that support the findings of this study are available from the corresponding author upon reasonable request.

## Supplementary Materials

The following supporting information can be downloaded at: https://www.mdpi.com/article/10.3390/antiox14040499/s1, Table S1. Primer sequences for qRT-PCR. Table S2. Quality control for RNA-seq data. Figure S1. qRT-PCR validation for RNA-seq results (n = 3). X-axis indicated the gene name while Y-axis represented the log2 (Fold change) value of each DEG. Relative expression levels were calculated based on 2^−ΔΔCT^ method.

## Appendix A. cDNA Library Construction and Transcriptome Sequencing

A total of 80 mg of fresh liver and gill tissue samples was collected, and total RNA was extracted using a standard TRIzol reagent (Invitrogen, Carlsbad, CA, USA) according to the manufacturer’s protocol. The quality (purity and concentration) of RNA was assessed using a NanoDrop ND-1000 spectrophotometer (Thermo Scientific, Wilmington, DE, USA). RNA degradation and contamination were determined based on 1% agarose gel electrophoresis. The qualified RNA samples were used for the construction of the sequencing library. Messenger RNA (mRNA) was enriched using oligo (dT) beads and cleaved into 150 bp fragments. Then, first-strand cDNA was synthesized using dNTP, RNase H, DNA polymerase I, and buffer. Following purification and end-repair, cDNA fragments were subjected to paired-end sequencing based on the Illumina HiSeqTM2500 platform (Allwegene Tech Co., Ltd., Beijing, China).

## Appendix B. Quantification and Differentially Expressed Gene (DEG) Analysis

The mapped reads of each sample were assembled based on the reference genome using the StringTie tool and then normalized to fragments per kilobase of transcript per million (FPKM). Clean data were analyzed using DESeq2 to identify the DEGs between the control and low/high groups. A Q value < 0.005 and |log2(fold change)| ≥ 1 were regarded as the thresholds for DEG filtration. Gene Ontology (GO) enrichment and Kyoto Encyclopedia of Genes and Genome (KEGG) pathway enrichment analyses were conducted using R based on the hypergeometric distribution, with GO terms and KEGG pathway annotations performed using GOseq R packages and KOBAS [29], respectively.

## Appendix C. Quantitative Real-Time PCR (qRT-PCR) Verification

To validate the accuracy of the sequencing results, typical DEGs (AG vs. DG, BG vs. DG, AS vs. DS, and BS vs. DS) were selected for comparison via qRT-PCR verification. Gene-specific primers were designed using Primer Premier 6.0 and synthesized by Sangon Co., Ltd., Shanghai, China (Table S1). The relative mRNA expression level was normalized based on the expression of the β-actin gene. First-strand cDNA was synthesized using a Prime ScriptTM RT reagent kit with DNA Eraser (TaKaRa, Kyoto, Japan). The reaction mixture contained 0.4 μL of each forward and reverse primer, 5 μL of 2 × TB Green Premix Ex Taq II (Tli RNaseH Plus), 3 μL of sterile distilled H2O (dH_2_O), 0.2 of μL 50 × ROX Reference Dye II (Roche, Basel, Switzerland), and 1 μL of template cDNA. qRT-qPCR analysis was performed using an ABI 7500 real-time PCR instrument (Thermo Fisher Scientific). The specific cycle parameters were established at 95 °C for 180 s, 40 cycles of 95 °C for 5 s, 60 °C for 15 s, and 72 °C for 30 s. The 2^−ΔΔCT^ method was employed for the calculation of the relative gene expression levels [30].

## Appendix D. Untargeted Metabolomics Analysis

Frozen liver and gill samples were thawed at 4 °C, and 55 mg of the sample was transferred into Eppendorf (EP) tubes and mixed with 500 μL of extraction solution (methanol/acetonitrile/water = 2:2:1). Liver and gill samples were homogenized at 35 Hz for 4 min and sonicated for 5 min in an ice–water bath. Then, the sample was centrifuged at 13,800× *g* for 15 min at 4 °C. Finally, the supernatant was transferred to a fresh glass vial for ultra-high-performance liquid tandem chromatography mass spectrometry (UHPLC-MS-MS) analysis. At the same time, liver and gill specimens were used to prepare quality control (QC) samples for methodological investigation. The raw UHPLC-MS-MS data were converted to mzXML format using ProteoWizard and processed with R and XCMS for peak detection, extraction, alignment, and integration. R package and AllwegeneDB tools were applied for metabolite identification. To analyze the differences between the control, low-ammonia, and high-ammonia groups, MetaboAnalystR was used to conduct an orthogonal partial least squares discriminant analysis (OPLS-DA) and principal component analysis (PCA). The metabolites with a VIP > 1, *p* < 0.05, and fold change less than 0.67 or more than 1.5 were defined as differential metabolites. In addition, KEGG (http://www.kegg.jp, 24 April 2024) and MetaboAnalyst (http://www.metaboanalyst.ca/, 24 April 2024) were utilized to identify signal pathway enrichment.

## Appendix E. 16S Ribosomal DNA Sequencing and Gut Microbiome Analysis

Fresh gut contents were collected using sterile scissors and forceps and transferred to RNase-free tubes. A MagPure Soil DNA KF Kit (Magen, Guangzhou, China) was used to extract total microbial DNA from the intestinal content, and the quantity of DNA was assessed utilizing a NanoDrop 2000 spectrophotometer (Thermo Fisher Scientific, USA). The hypervariable V3–V4 region of the 16S rRNA gene was amplified using a PCR thermocycler system (580BR10905, Bio-Rad, Hercules, CA, USA) with the 343F (5’-TACGGRAGGCAGCAG-3’) and 798R (5’-AGGGTATCTAATCCT-3’) primers. The PCR products were purified and quantified with AMPure XP beads (Agencourt, Beckmancoulter, CA, USA) and a Qubit dsDNA Assay Kit (Thermo Fisher Scientific, USA), respectively. According to the standard protocols of the OE Biotech Company (Shanghai, China), purified amplicons were pooled in equimolar concentrations and paired-end sequenced using the Illumina NovaSeq 6000 platform (Illumina, San Diego, CA, USA).

The software finally produced the representative text and amplicon sequence variant (ASV) feature table. All representative reads were subjected to annotation and subsequent blasting against the Silva database (Version 138) using the q2-feature-classifier with the default parameters. A Venn diagram was employed to ascertain the number of unique and shared ASVs in multiple samples. The differential intestinal microbiota of the low-ammonia group vs. control group and high-ammonia group vs. control group were determined. The alpha diversity was calculated using QIIME2 (2020.11), including Shannon, Chao1, and Simpson indices. The beta diversity was calculated via principal coordinate analysis (PCoA) plots based on unweighted UniFrac metrics using the R package. Utilizing PICRUSt2 (2.3.0b0), we predicted the COG and KEGG functions from the 16 S rDNA sequences.
