# Supplementary material for: Differential Cell Death Pathways Induced by Oxidative Stress in Multi-Organs of Amur Grayling (Thymallus grubii) Under Gradient Ammonia Stress
Source: Antioxidants (Basel). 2025 Apr 21;14(4):499. doi: 10.3390/antiox14040499 (PMC12023975; doi:10.3390/antiox14040499)
Supplement: Supplementary file 1 [file antioxidants-14-00499-s001.zip › antioxidants-3530716-supplementary.pdf]

Table S1. Primer sequences for qRT-PCR

Gene	Primer sequence	Product Length (bp)	Annealing temperature (℃)
Mmp17	F:CTGAGTATGTGGCGGGTCA	232	57.3
	R:CCCTCTCCAATGTCTGCCC		58.8
vtg	F:GTGTGGGCAGACATTGGAGAGA	221	59.6
	R:TGAGGGCAGTCCAACCAATC		57.8
Mmp17	F:CTGAGTATGTGGCGGGTCA	232	57.3
	R:CCCTCTCCAATGTCTGCCC		58.8
Lama1	F:CTTCATAGACCTGCACACGG	245	55.9
	R:CCACCAGCCTCCTCTCAC		58.7
nfm	F:GTGAAACGAACAGCAGTGACA	111	55.6
	R:CAGATGCCTTGGTCTTCACG		56.4
Tip41	F:TACAACAAAACACAGGGCAGG	146	55.6
	R:ATTCAGCCAACCACCCATAC		55.1
Shop21	F:GCTGAAGCAAGTGTTGGG	183	54.6
	R:TCCCGTCCTTCTCTGTGTCT		57.8
Gimap7	F:TCAGCCTTGTCTCAGGGTAG	244	56.4
	R:CGCCCTTTGTTCGATCTTGG		56.9
nin	F:GGAGCATTTCAGGAAGGAGAA	234	54.5

	R:GGTACAACAGTGCGTTGCT		56
Adipor2	F:TGCCGACCAACCTCTCTTTC	121	57.6
	R:GGGGGCAGTAAAGTGGTGAA		57.8

Table S2. Quality control for RNA-seq data

Sample name	Clean Reads	Clean Bases	Q20	Q30	GC Content
DG1	40617938	5.99G	98.10%	94.81%	47.80%
DG2	48226266	7.11G	97.96%	94.43%	47.56%
DG3	44756416	6.62G	97.87%	94.24%	47.79%
AG1	53264626	7.87G	97.95%	94.43%	48.03%
AG2	55864590	8.26G	97.81%	94.10%	48.09%
AG3	48459298	7.17G	97.90%	94.32%	48.15%
BG1	44772822	6.61G	98.18%	94.92%	46.28%
BG2	38497002	5.67G	97.83%	94.19%	46.76%
BG3	41727386	6.15G	98.14%	94.85%	46.68%
DS1	42693060	6.33G	97.61%	93.73%	45.60%
DS2	56283364	8.28G	97.59%	93.72%	45.51%
DS3	44497668	6.59G	97.59%	93.70%	45.38%
AS1	47860826	7.07G	97.75%	94.07%	46.42%
AS2	47798192	7.06G	97.75%	94.05%	46.25%
AS3	45625330	6.73G	97.74%	94.04%	46.14%
BS1	42396070	6.25G	97.86%	94.31%	44.53%
BS2	42490628	6.25G	97.63%	93.82%	43.78%
BS3	53084756	7.81G	97.96%	94.54%	44.69%

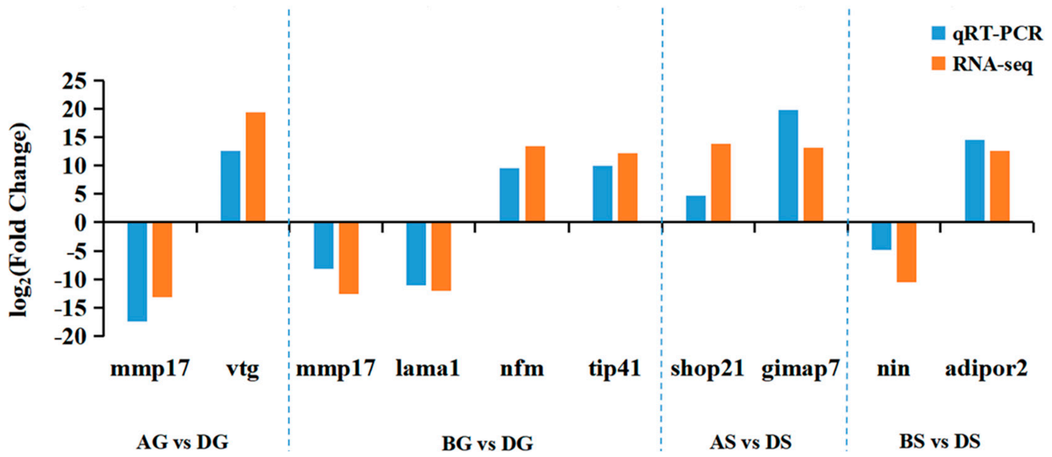

Figure S1. qRT-PCR validation for RNA-seq results (n=3). X-axis indicated the gene name while Y-axis represented the $\log_2$ (Fold change) value of each DEG. Relative expression levels were calculated based on $2^{-\Delta\Delta CT}$ method.
